# Downregulation of miR-1 in colorectal cancer promotes radioresistance and aggressive phenotypes

**DOI:** 10.7150/jca.44753

**Published:** 2020-06-07

**Authors:** Yong Wu, Ning Pu, Wenzhao Su, Xiaodong Yang, Chungen Xing

**Affiliations:** 1 Department of General Surgery, The Second Affiliated Hospital of Soochow University, Jiangsu, 215004, China.; 2 Department of General Surgery, Zhongshan Hospital, Fudan University, Shanghai, 200032, China.

**Keywords:** chemoradiotherapy, radioresistance, aggressive phenotypes

## Abstract

**Background:** Colorectal cancer (CRC) remains to be one of the most common malignancies worldwide. Various studies have demonstrated that microRNAs (miRs) play a critical role in regulating cancer progression and sensitivity to chemoradiotherapy. miR-1 was found to be aberrantly expressed in CRC. However, it has not been fully elucidated whether miR-1 regulated CRC cell radioresistance.

**Methods:** The expression of miR-1 was detected using quantitative real-time polymerase chain reaction in CRC tissues and cell lines. Colony survival and proliferation were determined using colony formation assay and MTT assay, respectively. Apoptosis and levels of related proteins, Bax and Bcl-2, were detected using flow cytometer assay and western blotting analysis. Migration and invasion were measured using wound healing assay and transwell invasion assay. The levels of invasion-associated proteins, E-cadherin, MMP2 and MMP9, were detected using western blotting analysis.

**Results:** miR-1 was found to be downregulated in CRC tissues and cell lines compared with adjacent normal tissues. *In vitro*, miR-1 overexpression significantly suppressed colony survival and proliferation, and induced cell apoptosis under irradiation, but no apoptosis was detected without irradiation. Furthermore, miR-1 mimics promoted the expression of Bax and E-cadherin and decreased the expression of Bcl-2, MMP2 and MMP9, and apparently impaired the invasion and migration of CRC cells in synergy with radiotherapy.

**Conclusion:** miR-1 enhanced the radiosensitivity of CRC cells by inducing cell apoptosis and the synergic inhibition of aggressive phenotypes, which may serve as a promising therapeutic target for CRC patients.

## Introduction

Despite innovations in therapy, colorectal cancer (CRC) remains to be one of the most common cancers worldwide and the third major cause of cancer-related deaths in the USA 1. Five out of one hundred individuals suffer from CRC worldwide, despite tremendous progress in treatment options available for CRC, including surgery, radiotherapy and chemotherapy, the 5-year survival rate remains at about 65% 2. Although surgery remains to be the main method of treatment for CRC patients, chemoradiotherapy plays an efficient role in advanced and metastatic diseases 3. However, radiotherapy and chemotherapy have been found to be associated with potential side effects and have failed in improving outcomes due to the occurrence of therapy resistance 4. Therefore, it is essential to improve the sensitization of CRC cells by developing technologies based on molecular markers.

MicroRNAs (miRs) are a new class of small noncoding (nc) RNAs of 18-22 bps in length, which play crucial roles in tumor biological behaviors through post-transcriptional regulation processes, such as apoptosis, proliferation, invasion and migration 5,6. miRs can act as oncogenes or tumor suppressor genes for the pathogenesis and progression of a variety of cancers 7. Several studies have reported that miRs regulate radiosensitivity of various types of cancers 8-10. For instance, Zheng et al. 8 found that miR-195 was downregulated in colorectal cancer cell lines, and that miR-195 overexpression could enhance the radiosensitivity of CRC cells by inducing apoptosis and suppressing colony survival through the suppression of CARM1 expression. Ectopic expression of miR-34a was found to have increased the radiosensitivity of non-small cell lung cancer cells, partially through the suppression of LyGDI expression 11.

miR-1 was first described as a muscle-specific miRNA 12. Several studies have found that the expression of miR-1 is downregulated in human cancers, including gastric cancer, lung cancer, hepatocellular carcinoma and bladder cancer, while the ectopic expression of miR-1 was found to have resulted in tumor suppression 13-16. Taniguchi et al. 17 reported that miR-1 showed a relatively low expression in CRC tissues, compared with that of adjacent normal tissues. Restoration of miR-1 levels inhibited epithelial-mesenchymal transition (EMT) by regulating the MAPK and PI3K/AKT pathways in CRC 18. Moreover, Jin et al. 19 illustrated that MALAT1 regulated nasopharyngeal carcinoma pathobiological behavior and radioresistance through the miR-1/Slug axis, indicating that miR-1 plays a potential role in inducing the radiosensitivity of cancer cells. The objective of this research study was to explore the effect of miR-1 on the radiosensitivity of CRC.

## Materials and methods

### Cell culture and transfection

A normal intestinal epithelial cell line (FHC) and established human colorectal carcinoma cell lines (HCT116, CCL244, SW480, HT29 and Lovo) were obtained from the Shanghai Institute of Cell Biology (Shanghai, China) and were cultured in DMEM (HyClone, Logan, UT, USA), supplemented with 10% fetal bovine serum (FBS; HyClone) and 1% Penicillin-Streptomycin solution (Beyotime, Shanghai, China). All cell lines were incubated at 37 °C in a humidified incubator with a 5% CO_2_ atmosphere.

The cells were seeded into 6-well plates and transiently transfected with Lipofectamine 2000 (Invitrogen, San Diego, CA, USA) and miR-1 mimics or the miRNA mimic Negative Control (GenePharma, Shanghai, China), according to the manufacturer's instructions. Real-time PCR was performed to confirm the efficiency of transfection.

### Study population

53 pairs of primary CRC specimens and adjacent normal colorectal tissues were obtained from resected CRC patients at the Second Affiliated Hospital of Soochow University between 2013 and 2015. All specimens were stored at -80 °C for further experiments. The patients provided informed consent and this study was approved by the Ethics Committee of Soochow University.

### Quantitative real-time PCR

For miRNA quantitation, total RNA was extracted from cells and tissues using TRIzol reagent (Invitrogen) and was reverse transcribed using a reverse transcription kit (Thermo-Fisher, Waltham, MA, USA). Quantitative real-time polymerase chain reaction (qRT-PCR) was performed using a SYBR Green kit (Tiangen, Beijing, China) on an ABI7500 system (Applied Biosystems, Foster City, CA, USA). U6 was used as an endogenous standard to normalize the expression of miR-1. U6 was amplified using a reverse primer (5′-GGAACGCTTCACGAATTTG-3′) and a forward primer (5′-ATTGGAACGATACAGAGAAGATT-3′), while miR-1 was also amplified using a reverse primer (5′-CAGAGCAGGGTCCGAGGTA-3′) and a forward primer (5′-GCCACGATGGAATGTAAAGAAGT-3′).

### Colony formation assay

The cells were seeded into 6-well plates after transfection. Twenty-four hours later, the cells were irradiated using X-ray doses of 0, 2, 4, 6 and 8 Gy. After an additional 10 to 14 days of incubation, the cells were immobilized using 4% paraformaldehyde and stained with Giemsa. The colonies that contained at least 50 cells were counted. Plate efficiency was calculated as follows: (number of colonies/number of cells inoculated) × 100%. A multitarget-single hit model was applied for the analysis, which was performed using SPSS software.

### MTT assays

The cells were seeded into 96-well plates (5 × 10^3^ cells/well) after transfection. After 24 h, the cells were exposed to a single-dose of irradiation. After different durations of incubation, 20 μl of 0.5 mg/ml MTT solution (Solarbio, Beijing, China) was added into each well and the cells were incubated for 4 h at 37 °C. The supernatant solution was then removed and 200 µl of DMSO was added into each well. Absorbance of the supernatant solution was measured using a microplate reader (Bio-Rad, Hercules, CA, USA) at a wavelength of 490 nm after 15 min of vibration.

### Cell apoptosis assays

The ratio of apoptotic cells was determined using FITC Annexin V apoptosis detection kit (BD, San Diego, USA), as previously described 20. In brief, cells were exposed to a single-dose of X-ray after transfection. After forty-eight hours of incubation, the cells were harvested and centrifuged at 300g for 5 min. Then, the cells were washed twice using PBS and were resuspended in 100 µl of a binding buffer, followed by 15 min of incubation with propidium iodide (PI) and Annexin V- FITC. Another 400 µl of the binding buffer was then added to the cells and analysis was conducted using FACSVerse flow cytometer (BD).

### Migration/Invasion assays

For the wound healing migration assays, cells were seeded into 6-well plates and transfected after 24 h of incubation. After irradiation, the cells in each group were scratched using a pipette tip and washed twice with PBS. Cells were incubated with serum-free DMEM, and images were captured using a microscope (Olympus, Tokyo, Japan) at 0 h and 48 h.

For the transwell invasion assay, 3×10^4^ cells were seeded into a Matrigel-coated Transwell^®^ chamber (Corning Incorporated, Corning, NY, USA). The lower part of the chamber was supplemented with DMEM medium containing 10% fetal bovine serum. After 48 h of incubation, the matrigel was removed using a cotton swab. Cells that had invaded were fixed using 4% paraformaldehyde and stained with Giemsa. The number of invading cells was counted under a microscope.

### Western blotting assays

Total lysates of CRC cells were extracted after transfection and irradiation using a cell lysis buffer (Solarbio). The protein concentration was measured using a Bicinchoninic Acid assay (Beyotime). Equal amounts of protein were subjected to 10% polyacrylamide gels, followed by transfer onto a PVDF membrane (Millipore, Temecula, USA) and were blocked using TBST (100 mM Tris-HCl, 0.9% NaCl and 0.1% Tween 20; pH 7.5) containing 5% (w/v) skimmed milk. The proteins were probed using Bax, Bcl-2, E-cadherin, MMP2 and MMP9 antibodies under chemiluminescence.

### Statistical analysis

All the above mentioned experiments were repeated at least three times. All results were presented as mean ±standard error and analyzed using the Statistical Package for the Social Sciences v17.0 (SPSS Inc., Chicago, IL, USA) and GraphPad Prism 5 software (San Diego, CA, USA). Student's t test was used to analyze differences between two groups, while one-way analysis of variance was used to determine statistical significance among three or more groups. A p value of <0.05 was considered to indicate a statistically significant result. p-value markings: *p < 0.05, **p < 0.01 and ***p < 0.001.

## Results

### miR-1 was downregulated in CRC cell lines and tissues

**Figure 1:**
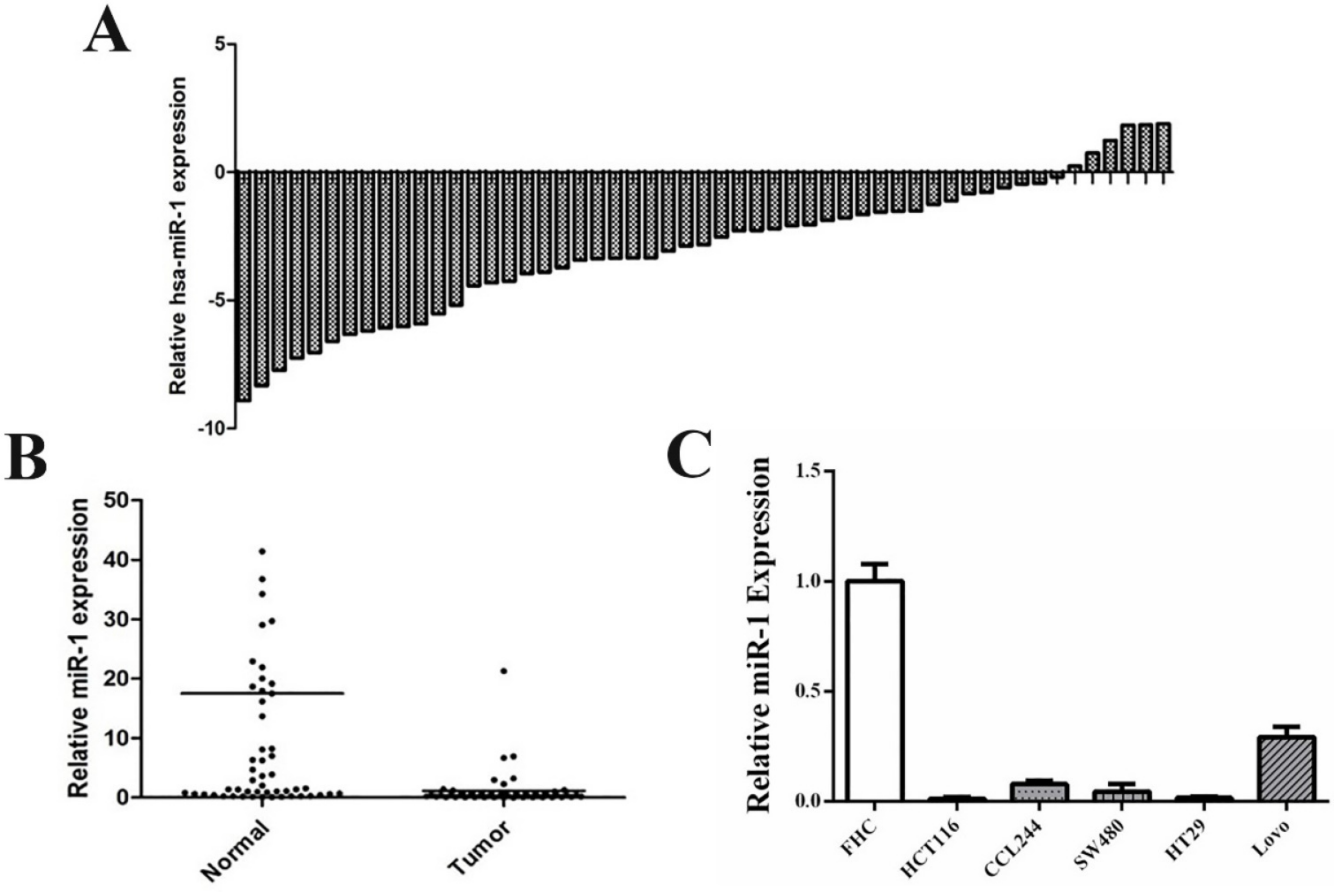
miR-1 expressions were down-regulated in CRC tissues and cell lines. (A) The distribution of miR-1 expression of CRC tissues in each case relative to that of its adjuvant normal tissues. (B) The comparison of miR-1 expression in CRC tissues and adjuvant normal tissues. (C) The miR-1 expression in CRC cell lines and normal cell line. CRC, colorectal cancer.

qRT-PCR was performed to detect the expression of miR-1 in resected CRC tissues and matched adjacent normal tissues (n=53). The results revealed that miR-1 expression in CRC tissues was lower than that of non-tumor tissues (**Figure 1A and B**). The clinicopathological characteristics of the enrolled patients are shown in **Table 1**. Next, we detected the expression level of miR-1 in a normal colonic mucosal cell line (FHC) and CRC cell lines (HT29, HCT116, CCL244, SW480 and Lovo), which showed that miR-1 expression was significantly downregulated in CRC cell lines, compared with that of the FHC cell line (**Figure 1C**). These results indicated that miR-1 may be involved in the tumorigenesis and progression of CRC. CCL244 has been confirmed as the most radioresistant cell line, followed in order by SW480, HT29, Lovo and HCT116, through our previous study 21. Therefore, CCL244 was selected to be used in the following studies to explore the role of miR-1 in CRC radioresistance.

### miR-1 upregulation sensitized CCL244 cells to radiation

**Figure 2:**
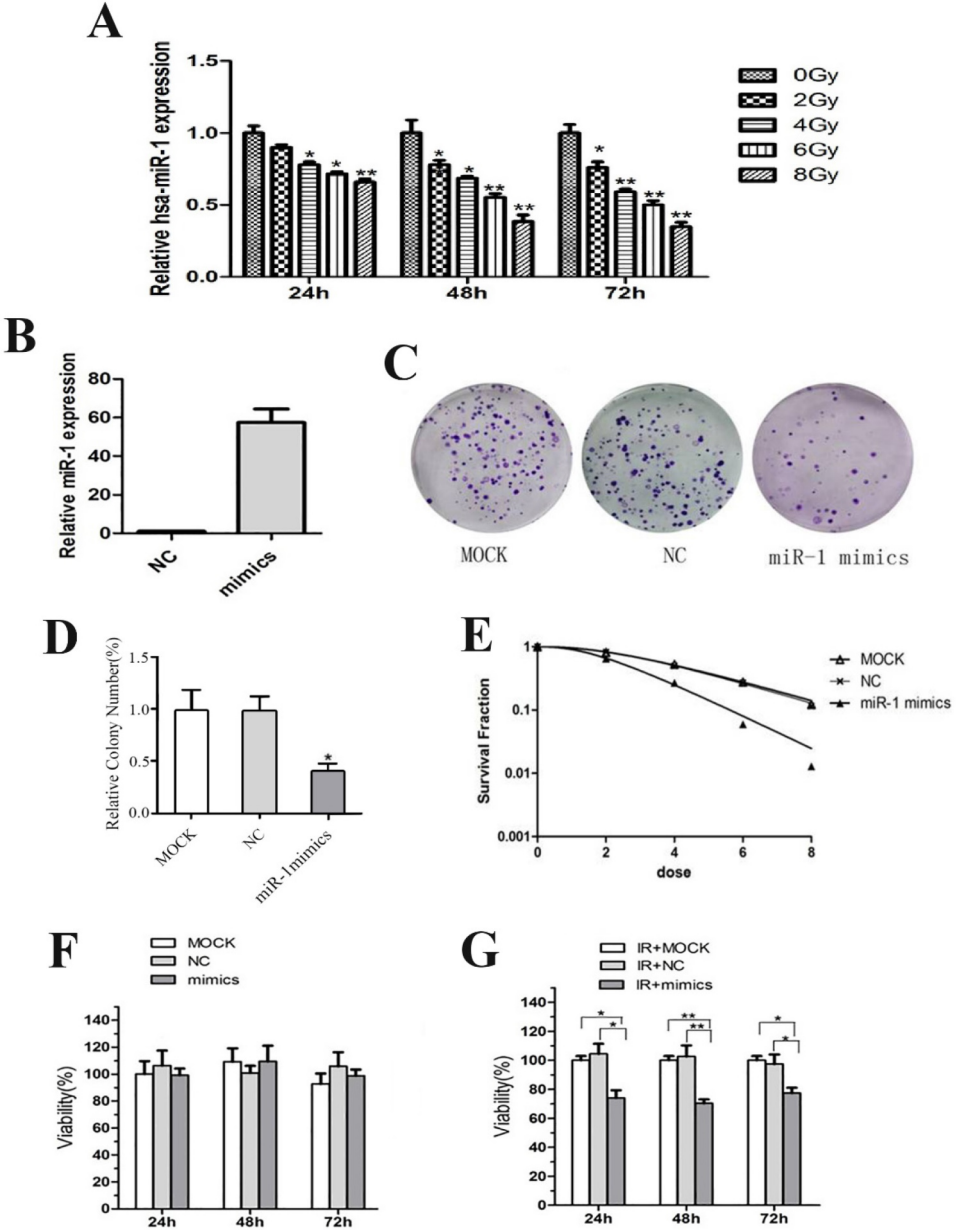
miR-1 enhanced the radiosensitivity of CCL244 cells. (A) The expression of miR-1 in a dose-dependent and time-dependent manner. (B) qRT-PCR detecting the efficient transfection of miR-1 mimics and negative control. (C, D) Colony formation assay and analysis showing the impact of miR-1 mimics on survival after irradiation. (E) Colony survival fraction at dose (2, 4, 6 and 8 Gy) dependent manner compared to MOCK and NC group. (F) The effect of miR-1 overexpression on proliferation of CCL244 cells at 0-Gy. (G) The effect of miR-1 overexpression on proliferation of CCL244 cells at 4-Gy irradiation. NC, negative control; *, p<0.05; **, p<0.01.

CCL244 cells were exposed to X-ray doses of 0, 2, 4, 6 and 8 Gy. After 24 h, 48 h and 72 h, total RNA was extracted and purified from CCL244 to detect the differential expression of miR-1. The results revealed that the expression of miR-1 decreased in a dose-dependent and time-dependent manner (**Figure 2A**). CCL244 cells were transiently transfected with the miR-1 mimics and miRNA negative control (NC) for use in the following experiments. The efficient transfection of miR-1 mimics and NC was confirmed using qRT-PCR (**Figure 2B**).

The colony formation survival assay is regarded as the standard in determining radiosensitivity 22. D0 values are used to indicate the radiosensitivity of cancer cells, with higher D0 values indicating higher resistance to radiation. The results of the colony survival assay showed that the miR-1 mimics could significantly decrease levels of colony formation after irradiation (**Figure 2C and D**), and that the upregulation of miR-1 (D0=1.672) significantly decreased the colony survival fraction in a dose-dependent (2, 4, 6 and 8 Gy) manner, compared with the MOCK (D0=2.876) and NC (D0=2.821) (**Figure 2E**). Furthermore, MTT assay was performed to evaluate the viability of transfected cells treated with 0 Gy and 4 Gy of irradiation followed by 24 h, 48 h and 72 h of culture. The results showed that the overexpression of miR-1 had no effect on the proliferation of CCL244 cells at 0 Gy (**Figure 2F**). However, after exposure to 4 Gy of irradiation, the viability of CCL244 with miR-1 upregulation was significantly inhibited (**Figure 2G**). Taken together, these results demonstrated that miR-1 could enhance the radiosensitivity of CCL244 cells.

### Overexpression of miR-1 increased radiation-related apoptosis of CCL244

**Figure 3:**
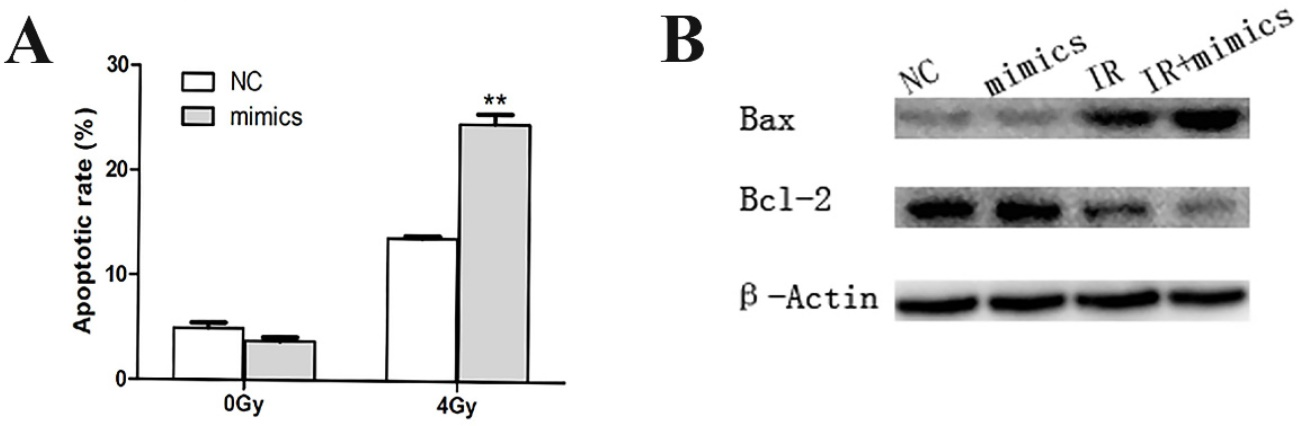
miR-1 up-regulation promoted irradiation-related apoptosis of CCL244. (A) Flow cytometer analysis showing apoptotic rate in the combination of miR-1 mimics and 4 Gy irradiation compared to irradiated NC group. (B) Western blotting analysis showing the expression of apoptotic-related proteins Bax and Bcl-2 in the combination treatment of miR-1 mimics and 4 Gy radiation. NC, negative control; **, p<0.01.

Apoptosis is the main method of cell death caused by radiotherapy. Flow cytometry was applied to detect the apoptotic rate of CCL244 cells pretreated through transfection and irradiation. Our data revealed that the influence of miR-1 upregulation on non-irradiated CCL244 cell apoptosis was not obvious. However, the apoptotic rate significantly increased after the combination of the miR-1 mimics and 4 Gy irradiation, compared with the irradiated NC group (**Figure 3A**). On the other hand, the expression of apoptosis-related proteins, Bax and Bcl-2, showed no statistical differences between miR-1 mimics and NC with no irradiation, but Bax levels were obviously increased and Bcl-2 levels decreased when CCL244 cells were subjected to the combination treatment of miR-1 mimics and 4 Gy radiation (**Figure 3B**). These results suggested that miR-1 upregulation could indeed promote the irradiation-related apoptosis of CCL244.

### Overexpression of miR-1 promoted a nonaggressive phenotype of CCL244

**Figure 4:**
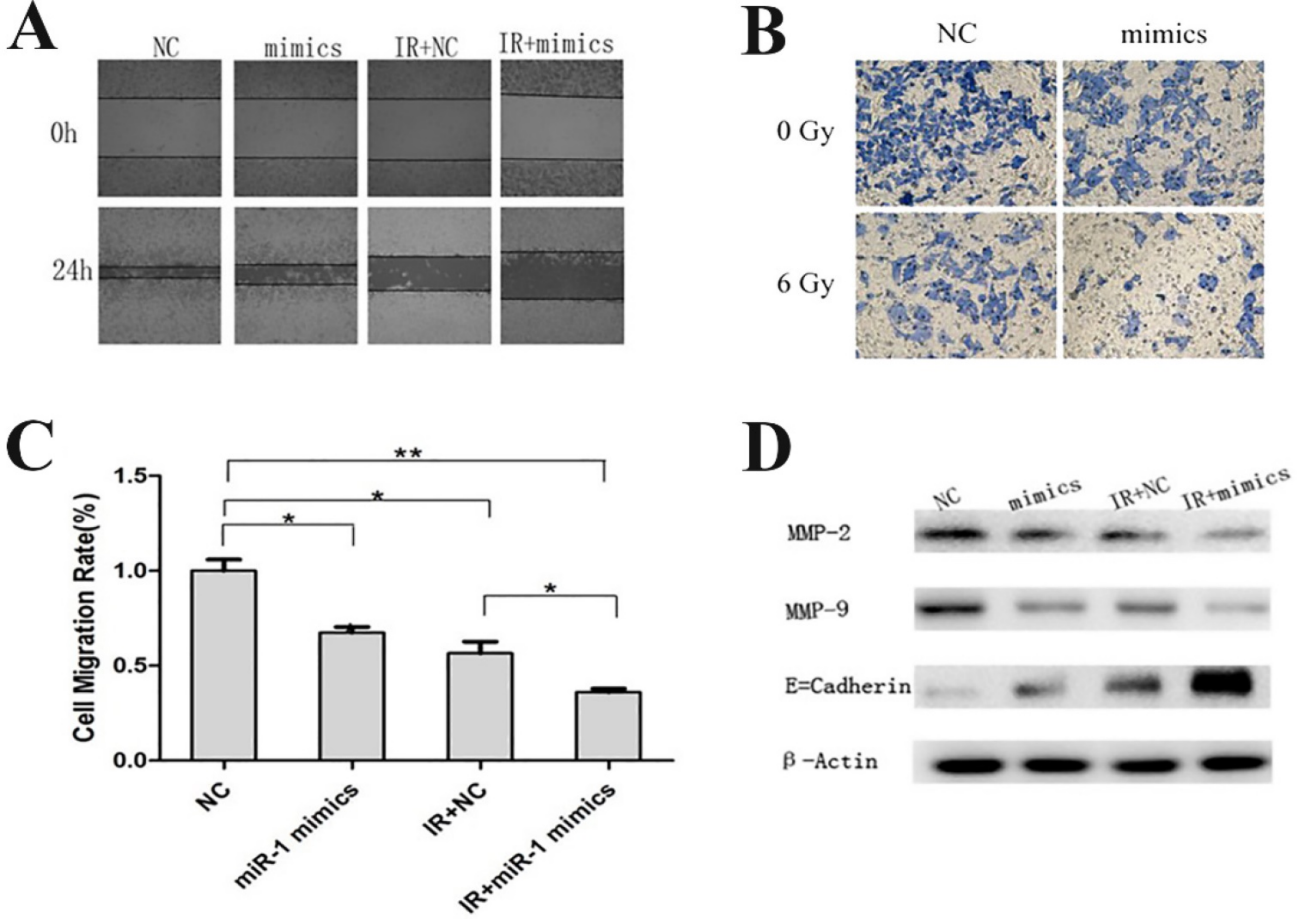
miR-1 up-regulation combined with radiotherapy impaired CCL244 invasion and metastasis. (A) Wound healing migration assay showing the impact of miR-1 on the ability of invasion with or without irradiation. (B, C) Transwell invasion assay and analysis showing the impact of miR-1 on the ability of metastasis with or without irradiation. (D) Western blot analysis showing the expression of metastasis-related proteins in the combination treatment of miR-1 mimics and radiation. *, p<0.05; **, p<0.01.

Invasion and metastasis are multistep processes that affect the prognosis of CRC patients. EMT is an important process during metastasis by which epithelial cells display mesenchymal cells properties and show increased motility 23. Wound healing migration assays and transwell invasion assays were performed to assess the effect of miR-1 on invasion and metastasis. Interestingly, with or without irradiation, the overexpression of miR-1 both impaired the potential of cell invasion and metastasis, and the inhibitory effect was more obvious under combination treatment (**Figure 4A-C**). Western blotting assays revealed that the expressions of metastasis-related proteins, matrix metalloproteinase 2 (MMP2) and MMP9, decreased and that the expression of epithelial marker E-cadherin increased when CCL244 cells were treated with miR-1 mimics or a combination of miR-1 mimics and radiation (**Figure 4D**). Based on the above results, we concluded that miR-1 upregulation combined with radiotherapy significantly impaired the invasion and metastasis of CCL244 cells.

## Discussion

Radiotherapy is the cornerstone of clinical treatment for CRC patients. Moreover, clinical evidence has suggested that neoadjuvant chemoradiotherapy is of value for the control of the local recurrence and improvement of the prognosis of rectal cancer 24,25. However, radioresistance is a critical challenge for patients receiving radiotherapy. Therefore, research studies are urgently required to identify the molecular mechanisms involved in radioresistance. Radioresistance may arise from multi-step processes, including the abnormal activation of DNA repair activity, microenvironmental hypoxia, aberrant survival pathway engagement, autophagy and angiogenesis 26-28. Recently, the dysregulated expression of miRNAs have been identified as independent prognostic factors that are associated with the radiosensitivity of various tumors 29. Moreover, miRNAs have been found to be involved in regulating CRC radioresistance 30. Our previous research revealed that the upregulation of miR-100 may sensitize CRC cells to X-ray radiation through the induction of apoptosis and DNA double-strand breaks 21. miR-31 can enhance the radiosensitivity of CRC cells by inhibiting autophagy in CRC associated fibroblasts 31. Recent studies have suggested that miR-1 may play an important role in CRC. The downregulation of miR-1 combined with that of MACC1 could increase the expression of MET and contribute to the metastasis of CRC 32. Furukawa et al. 33 demonstrated that miR-1-NOTCH3-Asef played a vital role in CRC cell migration. Considering that miR-1 dysregulation contributes to the pathological behavior of CRC, we investigated whether miR-1 was involved in the regulation of CRC radioresistance.

Previous studies have reported that miR-1 was found to be downregulated in most types of cancers 13-16, 34. Consistent with this finding, our data revealed that miR-1 downregulation was frequently found in CRC tissues and cell lines, compared with that of adjacent normal tissues, suggesting that miR-1 may play a tumor suppressive role in the development of CRC. Upregulation of miR-1 diminished tumor occurrence, the development and therapy resistance 35. Kim et al. 36 demonstrated that high-levels of miR-1 were associated with cisplatin/fluorouracil sensitivity in gastric cancer. However, knowledge regarding miR-1 function in CRC radioresistance is poor. Our results revealed that miR-1 was significantly downregulated along with an increase in radiation dose and time in CCL244 cells, which indicated its potentiality in regulating radiosensitivity. Moreover, the overexpression of miR-1 could increase the radiosensitivity of CCL244 cells, as evidenced by the decrease in the survival fraction.

It has been confirmed that miR-1 plays an important role in the proliferation, apoptosis and metastasis of many cancer types, including CRC 13-17, 33, 34. Our results showed that the proliferation ability and apoptosis rate of CCL244 cells remained unchanged following the overexpression of miR-1. However, miR-1 upregulation in combination with radiotherapy could significantly impair proliferation and induce the synergic apoptosis of CCL244 cells. It has been well-documented that radiation-resistant cancer cells possess an anti-apoptosis capacity 19. Ke et al. documented that radioresistant cervical carcinoma showed high miR-181a expression levels 37. miR-181 negatively regulated levels of the pro-apoptotic protein kinase, PRKCD, which resulted in the inhibition of irradiation-induced apoptosis. Similarly, the overexpression of miR-126 was found to have sensitized non-small cell lung cancer cells to irradiation-induced apoptosis through the PI3K/Akt pathway 38. Therefore, sufficient comprehension of the apoptotic mechanism is essential for overcoming therapy resistance 39. Given that miR-1 up-regulation could enhance radiation-induced apoptosis, we further examined the expression of apoptosis-related proteins. We found that miR-1 upregulation increased the expression of the pro-apoptotic protein, Bax, and decreased the expression of the anti-apoptotic protein, Bcl-2, following radiotherapy. Taken together, we concluded that miR-1 could modify the radiosensitivity, partially through the induction of apoptosis.

Cell motility is a multi-step process requiring the stimulation of many cellular processes 40. EMT is induced via various alterations, including loss of basal apical polarity, cell adhesion and gain of motility 41. E-cadherin levels have been found to have decreased during EMT, whereas N-cadherin expression was elevated, resulting in a cadherin switch 42. miR-1 expression could restore E-cadherin expression in order to increase cell-cell contacts through the formation of adherent junctions 43, 44. MMPs, a family of endopeptidasesare, are found in the extracellular environment and are responsible for the degradation of extracellular matrix 45. Thus, MMPs play a pivotal role in the invasiveness and metastasis of various cancers. In this study, we performed invasion-related assays to evaluate the role of miR-1 expression and radiotherapy on CRC cells. We found that miR-1 upregulation impaired the invasion and metastasis of CCL244 cells, and this effect was more pronounced in combination with radiation therapy. In addition, western blotting assays revealed that miR-1 upregulation combined with radiotherapy triggered the significant upregulation of E-cadherin and downregulation of MMP2 and MMP9. Taken together, these results indicated that miR-1 could enhance the radiosensitivity of CRC cells through the synergic inhibition of invasion and metastasis.

Although we found that miR-1 enhanced the radiosensitivity of CRC cells by inducing cell apoptosis and synergic inhibition of aggressive phenotypes, which may serve as a promising therapeutic target for CRC patients our study has several limitations, so that the use of *in vivo* models is warranted to validate our findings and a higher number of clinical cases are needed to determine the clinical value of miR-1 in predicting sensitivity to radiotherapy.

## Conclusion

In summary, the expression of miR-1 was found to be downregulated in CRC tissues and cell lines. Enforced expression of miR-1 enhanced the radiosensitivity of CRC cells by inducing cell apoptosis and synergic inhibition of aggressive phenotypes. Although the relevant mechanisms by which miR-1 effects radiosensitivity remain unclear and need to be further studied, our data suggested that miR-1 might serve as a promising therapeutic target for CRC patients with radioresistance.

## Figures and Tables

**Table 1:** The clinicopathological characteristics of colorectal cancer patients in this study

Parameters	Numbers
Cases	53
**Age**	
<65	30
≥65	23
**Tumour size**	
Small size (<5 cm)	28
Large size (≥5 cm)	25
**Gender**	
Male	35
Female	18
**Invasion levels**	
Mucosa	4
Submucosa	3
Muscle	10
Serosa	36
**TNM stage**	
Stages 1-2	34
Stages 3-4	19
**Lymph node metastasis**	
Positive	26
Negative	27
**Grade of tumors**	
Low grade	13
Intermediate grade	33
High grade	7
**Vascular invasion**	
Positive	22
Negative	31
**Perineural invasion**	
Positive	17
Negative	36

## Abbreviations

CRC: colorectal cancer
miR: microRNA
EMT: epithelial-mesenchymal transition
FBS: fetal bovine serum
qRT-PCR: quantitative real-time polymerase chain reaction
PI: propidium iodide
NC: negative control
MMP: matrix metalloproteinase
